# Geo-mapping of time trends in childhood caries risk a method for assessment of preventive care

**DOI:** 10.1186/1472-6831-12-9

**Published:** 2012-06-11

**Authors:** Ulf Strömberg, Anders Holmn, Kerstin Magnusson, Svante Twetman

**Affiliations:** 1 Department of Research and Development, Halland Hospital, SE-301 85, Halmstad, Sweden; 2 Department of Occupational and Environmental Medicine, Lund University, SE-221 85, Lund, Sweden; 3 Section of Community and Preventive Dentistry, Maxillofacial Unit, Halland Hospital, SE-301 85, Halmstad, Sweden; 4 Department of Cariology, Endodontics, Pediatric Dentistry and Clinical Genetics, Institute of Dentistry, Faculty of Health and Medical Sciences, University of Copenhagen, Nrre All 20, 2200, Copenhagen N, Denmark

**Keywords:** Caries, Children, Prevention, Geo-mapping, Time trend

## Abstract

**Background:**

Dental caries is unevenly distributed within populations with a higher burden in low socio-economy groups. Several attempts have been made to allocate resources to those that need them the most; there is a need for convenient approaches to population-based monitoring of caries risk over time. The aim of this study was to develop the geo-map concept, addressing time trends in caries risk, and demonstrate the novel approach by analyzing epidemiological data from preschool residents in the region of Halland, Sweden.

**Methods:**

The study population consisted of 9,973 (2006) and 10,927 (2010) children between 3 to 6years of age (~77% of the eligible population) from whom caries data were obtained. Reported dmfs>0 for a child was considered as the primary caries outcome. Each study individual was geo-coded with respect to his/her residence parish (66 parishes in the region). Smoothed caries risk geo-maps, along with corresponding statistical certainty geo-maps, were produced by using the free software Rapid Inquiry Facility and the ESRI ArcGIS system. Parish-level socioeconomic data were available.

**Results:**

The overall proportion of caries-free (dmfs=0) children improved from 84.0% in 2006 to 88.6% in 2010. The ratio of maximum and minimum (parish-level) smoothed relative risks (SmRRs) increased from 1.76/0.44=4.0 in 2006 to 2.37/0.33=7.2 in 2010, which indicated an increased geographical polarization of early childhood caries in the population. Eight parishes showed evidential, positional changes in caries risk between 2006 and 2010; their corresponding SmRRs and statistical certainty ranks changed markedly. No considerable parallel changes in parish-level socioeconomic characteristics were seen during the same time period.

**Conclusion:**

Geo-maps based on caries risk can be used to monitor changes in caries risk over time. Thus, geo-mapping offers a convenient tool for evaluating the effectiveness of tailored health promotion and preventive care in child populations.

## Background

The prevalence of dental caries has declined globally over the past decades but all children have not benefited from the improved oral health. Widening inequalities between social classes and certain minority ethnic groups are evident [1-5]. Caries risk assessment (CRA) is an essential component in the decision-making process for the prevention and management of the disease [6]. For individuals, background data on host factors, diet and oral hygiene are commonly merged with findings from a clinical examination while CRA in populations most often rely on epidemiological data and/or socioeconomic determinants. An increased caries risk should preferably be linked to intensified preventive and non-operative care and many such initiatives have been launched globally [7-9]. However, the lack of convenient and effective tools to monitor the outcome of such health promoting activities has limited the evaluation of effectiveness. There is a need for new approaches to population-based monitoring of caries risk over time.

In a recent paper we suggested the use of geo-maps for presenting epidemiological data based on childhood caries risk in a Swedish population and we advocated this novel approach for allocation of preventive care [10]. In order to evaluate the effect of preventive measures on childhood caries, there is a need for a method that compares repeated geo-maps. In the present communication, the geo-map concept is developed to address time trends in caries risk. We demonstrate the novel approach by analyzing and examining time trends in childhood caries risk (3-6years) based on epidemiological data from two occasions over four years in the southwest Swedish region of Halland.

## Methods

### Study population

The vast majority of all children (around 90%) in the region are listed as regular patients at the Public Dental Service that provides free dental care between 1 and 19years with recall intervals varying from 3 to 24months depending on the individual need. Data on the experience of manifest (dentin) caries is registered according to the WHO-criteria [11] and annually reported to the community dentistry unit. The fluoride concentration in piped water supply is generally low (<0.3ppm) except in the northern part (the municipality of Kungsbacka) where the natural fluoride content in the drinking water is approximately 1.0ppm.

The present study included 9,973 children between 3-6years of age for whom caries data were reported in 2006 and 10,927 children with corresponding data from 2010. The age distributions of the children examined in 2006 and 2010, respectively, did only differ marginally (proportions of children aged 5years: 49.3% in 2006; 50.3% in 2010). The overall coverage of the total 3-6-year population of the Halland region was 76% in 2006 and 77% in 2010. The remaining children were not recalled for a regular check-up that particular year or visited a private dentist at a clinic not among the in-reporting ones or located outside the region. The study was approved by the Halland Hospital Ethical committee as well as The Swedish Data Inspection Board.

### Geographical information system (GIS) methods

The Halland region consists of six municipalities that are subdivided into 66 parishes. Geo-maps were produced by using the ESRI ArcGIS system (Environmental Systems Research Institute, Inc., USA). Each child was geo-coded with respect to his/her residence area (parish).

### Socio-economic characteristics

Statistics Sweden provided parish-level data from years 2006 and 2010, respectively, on three socio-economic indicators: (i) the proportion with post-secondary education (any schooling beyond the high-school level) among all residents; (ii) the proportion of immigrants (more specifically, individuals born outside Sweden and individuals born in Sweden with both parents born outside Sweden) among all residents; and (iii) the proportion of families with low purchasing power (according to Swedish standard; 19,500 USD annual income) among all residing families with at least one child (19 years old; family with the same residence address).

### Epidemiological and statistical methods

Reported dmfs>0 for a child was considered as the primary caries outcome. The method for estimating caries risks at a given year of reporting has been explained previously [10]. Briefly, a parish-level relative risk (RR) was calculated as the observed-to-expected ratio, where the expected number was obtained from the sex-specific caries (dmfs>0) rates for the total study population of 3-6year old children residing in the Halland region. Moreover, *smoothed* RRs (SmRR) for each parish were obtained by running a Bayesian hierarchical mapping model, which allow parish-specific RRs to be smoothed towards global and local average risk levels across the study region [12]. We underline that such Bayesian smoothing yielded shrinkage of the conventional observed-to-expected ratios. The corresponding statistical certainty geo-maps were obtained by calculating the posterior probabilities of a parish-specific relative risks above 1 given the data, denoted Pr(RR>1|data), using the Bayesian approach. A parish with data yielding strong statistical evidence of an elevated caries risk, more precisely Pr(RR>1|data)>0.95, was colored *red* in the certainty geo-map. By contrast, a parish with evidently lowered caries risk, Pr(RR<1|data)=1 - Pr(RR>1|data)>0.95, was colored *green*. The remaining parishes were colored *yellow*, which indicates a weaker statistical evidence for an elevated/lowered relative risk.

*Evidential, positional* changes in the caries risk estimated for the children living in a specific parish in 2006 and 2010, respectively, were assessed by comparing the certainty geo-maps along with the posterior probabilities, Pr(RR>1|data): if the certainty color changed and Pr(RR>1|data) differed at least 25% for a specific parish, when comparing the results for the study years 2006 and 2010, the positional change was considered evidential. Hence, the rationale for classifying a positional change as evidential is based on the change over time in the *statistical evidence* for an elevated (or lowered) relative risk. Clearly, it seems extraordinary to observe a parish with a positional change from an evidently elevated caries risk (red) in 2006 to an evidently lowered caries risk (green) in 2010, or vice versa. Nevertheless, there were more modest, yet substantial, positional changes (from red/green to yellow, or vice versa) that we considered evidential. Also notice that a parish could change certainty color without being indentified to have an *evidential* positional change (provided Pr(RR>1|data) differed less than 25%).

The statistical analyses were performed using the free software Rapid Inquiry Facility [13], which provides an extension to ESRI ArcGIS functions [14], along with free software for Bayesian data analyses, WinBUGS [15].

## Results

**Figure 1:**
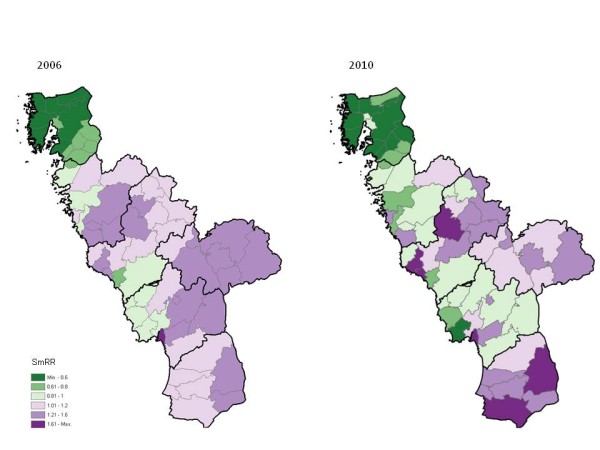
**Geo-maps of caries risk in 3-6-years old children.** Caries risk geo-maps of the Halland region (southwest Sweden) in 2006 and 2010, respectively, displaying, for each of the 66 residential parishes, the smoothed relative risk (SmRR) of caries (dmfs>0) among preschoolers (3-6years). The thicker borderlines delimit the six municipalities of Halland. The study population in year 2006 included 9,973 children; 84.0% caries-free (dmfs=0). The study population in year 2010 included 10,927 children; 88.6% caries-free. The ratio between the maximum and minimum SmRRs (considering the 66 parish-specific SmRRs) was 1.76/0.44=4.0 in 2006; 2.37/0.33=7.2 in 2010.

**Figure 2:**
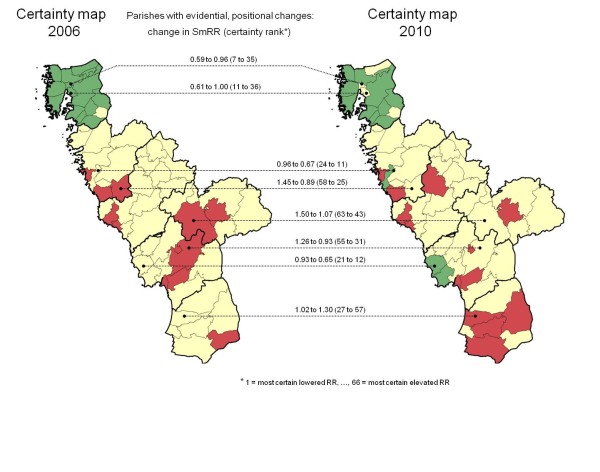
**Certainty geo-maps of elevated/lowered relative risk of caries in 3-6-years old children; parishes with evidential, positional changes in caries risk indicated with connecting lines.** Statistical certainty geo-maps of the Halland region (southwest Sweden) in 2006 and 2010, respectively, for each of the 66 residential parishes [*red color*, Pr(RR>1|data)>0.95, i.e. a parish with data yielding strong statistical evidence of an elevated caries (dmfs>0) risk; *green color*, Pr(RR<1|data)>0.95, i.e. a parish with data yielding strong statistical evidence of a lowered caries risk; and *yellow color*, a parish with data yielding weaker statistical evidence for a high or low relative risk]. The thicker borderlines delimit the six municipalities of Halland. The eight parishes with evidential, positional changes in caries risk are indicated with connecting lines providing information on changes in the smoothed relative risk (SmRR) and certainty rank (between 2006 and 2010).

The geo-maps of early childhood caries risk in 2006 and 2010, respectively, are presented in Figure 1. The overall proportion of caries-free (dmfs=0) children improved from 84.0% in 2006 to 88.6% in 2010. The geographical variation in caries risk was obvious in both years. The ratio between the maximum and minimum parish-level SmRRs increased from 1.76/0.44=4.0 in 2006 to 2.37/0.33=7.2 in 2010, which indicated accentuation of the geographical polarization of early childhood caries in the population. The corresponding statistical certainty geo-maps are presented in Figure 2. The eight parishes with evidential, positional changes in caries risk are indicated with connecting lines. For nine parishes, an evidently elevated relative risk in 2006 was weakened in 2010 (from red to yellow, n=4), or vice versa (from yellow to red, n=5). The positional changes in caries risk were considered evidential in four of those parishes. For six parishes, an evidently lowered risk in 2006 had weakened evidence in 2010 (from green to yellow, n=3), or vice versa (from yellow to green, n=3). The positional changes in caries risk were considered evidential in four of those parishes. There were marked changes in SmRRs as well as in certainty rank (1=most certain lowered relative risk; 66=most certain elevated relative risk) for the parishes showing evidential changes. For example, in a parish in the northern part of the region, with SmRR=0.59 and among the top-10 according to certainty rank in 2006 (262 study children; 90.5% caries-free; dmfs=0), the SmRR increased to 0.96 and the certainty rank fell to 35 in 2010 (308 study children; 88.0% caries-free).

For the eight parishes showing evidential positional changes in caries risk, no notable parallel changes in the parish-level socioeconomic characteristics were seen (Table 1). Although the proportion of immigrants increased over the time period, the rank positions of the parishes at issue were similar in 2006 and 2010, respectively (Table 1).

**Table 1:** Descriptive data for three socio-economic indicators on the parish-level

	2006	2010
*Socio-economic indicator*			*Range in the Halland region*
Parish^*^			Proportion in parish (rank)
*Proportion with post-secondary education among all residents (%)*	*10.4-48.4*	*10.0-48.3*
735	28.8 (53)	29.8 (54)
1136	25.6 (49)	25.1 (46)
2112	32.9 (60)	34.8 (59)
2411	32.6 (59)	35.1 (60)
2757	23.2 (42)	23.5 (42)
5531	22.6 (40)	22.9 (40)
5825	24.1 (46)	22.7 (39)
6343	15.0 (13)	15.0 (13)
*Proportion of immigrants among all residents (%)*	*1.9-30.7*	*4.0-33.9*
735	10.4 (52)	11.4 (50)
1136	6.7 (29)	7.4 (26)
2112	5.2 (13)	5.9 (9)
2411	5.7 (15)	6.7 (18)
2757	11.5 (55)	13.9 (55)
5531	7.5 (37)	8.8 (37)
5825	4.4 (4)	6.7 (16)
6343	12.9 (58)	15.3 (57)
*Proportion of families with low purchasing power among all residing families with at least one child ( 19years old) (%)*	*8.7-38.1*	*8.2-39.5*
735	20.2 (35)	20.9 (32)
1136	16.6 (21)	18.1 (24)
2112	15.2 (15)	14.2 (12)
2411	11.0 (6)	9.2 (4)
2757	22.0 (38)	22.0 (36)
5531	16.5 (20)	17.6 (21)
5825	29.0 (60)	29.3 (57)
6343	28.1 (58)	26.1 (49)

* Data for the eight parishes with evidential, positional changes in caries risk shown in Figure 2; identification for each parish is the certainty rank for relative risk in 20062010.

Rank in the Halland region (1=lowest proportion; 66=highest proportion).

## Discussion

We demonstrated that geo-maps based on caries risk can be used to monitor changes in caries risk over time. An important finding from the present study was that the geo-map concept was sensible enough for disclosing changes in geographical caries risk patterns among preschool children over a 4-year period, in spite of minor changes in absolute caries risk and stable contextual socioeconomic characteristics. We also underline that the Bayesian smoothing of the relative risks yielded essentially conservative estimates, meaning moderate sensitivity but high specificity [16]. The regression to the mean problem, i.e. the phenomenon that a by-chance elevated (or lowered) relative risk changed towards the null from one occasion to another, was thereby tackled.

The geo-mapping methodology proved to be rapid and robust albeit, as always, depending on the quality of the input data. The high coverage of data (>75%) indicates that the findings are valid for the entire population. There is always a risk that non-examined children or children of families avoiding dental visits have an impaired oral health but that risk was low in the present study groups. In fact, the dominating reason for not being examined was a prolonged recall interval (up to 18months) due to an estimated low caries risk [10].

The finding of an increased polarization of caries risk in spite of a generally improved dental health over the 4-year period was unwanted but not unexpected in the light of current papers on caries trends [17-20]. The Public Dental Service in the region is strongly encouraged to work actively with CRA and individual recall intervals. It should however be stressed that the overall cumulative caries burden in the present study population was low from an international perspective [21]. Nevertheless, the present time-trend geo-maps enable more detailed analyses for the possible local reasons for the certain changes in SmRRs on parish and/or clinic level. For example, the parish in the northern part of the region that fell from top-10 to the mid-range in four years has seen a rapid growth and urbanization with relocation of many young families. It is also important to realize that the ranking is relative. One parish with unchanged and low caries risk might be passed by one slightly improving its caries risk. Likewise, a decreased SmRR ranking might not necessarily mean that the caries risk de facto is significantly increased.

Our purpose was to disclose changes geographical patterns in childhood caries risk over time, without adjusting the parish-level relative risks (SmRRs) for contextual socioeconomic characteristics (which are geographically confounded factors [22]). If a parallel change in a socioeconomic determinant of parish-level relative risk occurs, further adjustments of the SmRR can provide insights to what extent the altered contextual position had an impact. However, we did not observe any considerable parallel changes in the available contextual socioeconomic indicators. Beyond the contextual socioeconomic data, we had lack of data on possible explanatory variables. Of course, it is essential to collect such data for planned assessments of preventive care. In fact, in the Halland region, there is a plan to collect data from the dental clinics regarding preventive care and, also, a proposal to reallocate resources for preventive care. Notwithstanding those planned efforts, we have demonstrated that our proposed method based on repeated geo-maps provides a useful assessment tool.

We are fully aware that there is limited evidence for methods to bridge inequalities in early childhood caries and that compliance for both self-applied and professional measures is a paramount concern [23]. However, for the preschool ages with adult responsibility, several controlled field trials have demonstrated that education, motivational interviews, coaching, tooth brushing and fluoride can significantly improve oral health and especially in vulnerable and deprived populations [24,25]. Geo-maps addressing time trends can hopefully be used to increase the understanding and improve the quality of follow-ups in future studies with the goal to optimize the strategies to eradicate early childhood caries. Indeed, our findings strongly indicate that repeated geo-maps over time could be useful to monitor the effect of population-based interventions as well as targeted programs for individuals with increased caries risk. Interestingly, feed-back on interventions and programs might be available in a relative short time span. The effectiveness of such programs is probably not limited to dentistry but can also be valid for current and future school-based activities such as promoting a healthy lifestyle and fighting over-weight and obesity in childhood. We reinforce the assumption that the common risk factor approach (high-sugar diet, fats, etc.) should be at focus for the prevention of both dental and medical diseases [10].

## Conclusion

Geo-maps based on caries risk can be used to monitor changes in caries risk over time. Thus, geo-mapping offers a convenient tool for evaluating effectiveness of tailored health promotion and preventive care in child populations.

## Competing interests

The authors declare that they have no competing interests.

## Authors contributions

All of the listed authors contributed to the conduct of the study. US, AH and ST analyzed and interpreted the data. US and ST drafted the manuscript. KE provided technical and administrative support. All authors approved the final version of this manuscript.

## Pre-publication history

The pre-publication history for this paper can be accessed here:

http://www.biomedcentral.com/1472-6831/12/9/prepub
